# Cryo-EM structure of mycobacterial cytochrome *bd* reveals two oxygen access channels

**DOI:** 10.1038/s41467-021-24924-w

**Published:** 2021-07-30

**Authors:** Weiwei Wang, Yan Gao, Yanting Tang, Xiaoting Zhou, Yuezheng Lai, Shan Zhou, Yuying Zhang, Xiuna Yang, Fengjiang Liu, Luke W. Guddat, Quan Wang, Zihe Rao, Hongri Gong

**Affiliations:** 1 Shanghai Institute for Advanced Immunochemical Studies and School of Life Science and Technology, ShanghaiTech University, Shanghai, China; 2 State Key Laboratory of Medicinal Chemical Biology, Frontiers Science Center for Cell Responses, College of Life Sciences, Nankai University, Tianjin, China; 3 CAS Center for Excellence in Molecular Cell Science, Shanghai Institute of Biochemistry and Cell Biology, Chinese Academy of Sciences, Shanghai, China; 4 University of Chinese Academy of Sciences, Beijing, China; 5 School of Chemistry and Molecular Biosciences, The University of Queensland, Brisbane, QLD Australia; 6 National Laboratory of Biomacromolecules, CAS Center for Excellence in Biomacromolecules, Institute of Biophysics, CAS, Beijing, China; 7 Laboratory of Structural Biology, Tsinghua University, Beijing, China

**Keywords:** Cryoelectron microscopy, Cryoelectron microscopy

## Abstract

Cytochromes *bd* are ubiquitous amongst prokaryotes including many human-pathogenic bacteria. Such complexes are targets for the development of antimicrobial drugs. However, an understanding of the relationship between the structure and functional mechanisms of these oxidases is incomplete. Here, we have determined the 2.8 Å structure of *Mycobacterium smegmatis* cytochrome *bd* by single-particle cryo-electron microscopy. This *bd* oxidase consists of two subunits CydA and CydB, that adopt a pseudo two-fold symmetrical arrangement. The structural topology of its Q-loop domain, whose function is to bind the substrate, quinol, is significantly different compared to the C-terminal region reported for cytochromes *bd* from *Geobacillus thermodenitrificans* (*G. th*) and *Escherichia coli* (*E. coli*). In addition, we have identified two potential oxygen access channels in the structure and shown that similar tunnels also exist in *G. th* and *E. coli* cytochromes *bd*. This study provides insights to develop a framework for the rational design of antituberculosis compounds that block the oxygen access channels of this oxidase.

## Abstract (web summary)

Cytochromes *bd* oxidase (Cyt-*bd*) catalyzes the reduction of oxygen to water and is the terminal oxidase in the respiratory chain of prokaryotes. Here, the authors present the 2.8 Å cryo-EM structure of *Mycobacterium smegmatis* Cyt-*bd* and identify two potential oxygen access channels in the structure, which is of interest for the development of novel antituberculosis drugs.

## Introduction

Respiratory oxygen reductases (terminal oxidases) comprise a series of structurally distinct enzymes that are widely distributed across all kingdoms of life. The heme-copper oxidases (HCO) and *bd*-type oxidases (cytochromes *bd*) are two well-known types of membrane-integrated terminal oxidases^1,2^. They catalyze the reduction of molecular oxygen (O_2_) to water by the respiratory substrate, cytochrome *c* or quinol, coupled to the generation of a proton motive force utilized for adenosine triphosphate (ATP) synthesis^3,4^. Compared to the well-characterized HCOs, cytochromes *bd* have not been widely studied. These cytochromes are only present in prokaryotes, which include many human pathogens, and thus belong to an evolutionarily distinct oxidase family^4^.

Cytochrome *bd* oxidases possess a high affinity for oxygen^5,6^, which facilitates bacterial survival under O_2_-poor environments^7,8^. Apart from this, cytochromes *bd* also endow bacteria with a number of vitally important physiological functions including enhancing tolerance to nitrosative stress^9^, contribute to resistance to hydrogen peroxide^10^, suppress extracellular superoxide production^11^, and confer the ability to defend against antibacterial agents^12^. It is likely that these properties of cytochromes *bd* promote virulence in a number of bacterial pathogens that cause serious infectious diseases to humans, such as *Mycobacterium tuberculosis*^13^, *Brucella abortus*^14^, as well as *Salmonella Typhimurium*^15,16^, *Bacteroides*^7^, and *Listeria monocytogenes*^17^. Since cytochrome *bd* is a key enzyme for the survival of prokaryotes and is absent in mammals, it is a promising therapeutic target for the development of antibacterial agents^4,18^.

To date, two structures of the *bd* oxidases have been reported. One is from *Geobacillus thermodenitrificans* (*G. th*)^19^ and the other is from *Escherichia coli* (*E. coli*)^20,21^. These enzymes possess two key subunits CydA and CydB but vary in the numbers of additional small subunits that are associated with them. *G. th bd* oxidase contains an association subunit called CydS and *E. coli bd* oxidase includes two association subunits named CydX and CydH (or CydY). Common features in these complexes are two *b*-type hemes (low-spin heme *b*_558_ and high-spin heme *b*_595_) and one *d*-type heme which are arranged in a triangular manner but with different relative positions in the two structures. These observations suggest that homologous *bd* oxidases share a similar architecture but can vary in mechanistic detail^21–24^. Therefore, more structural information is needed to obtain a comprehensive knowledge of the structure and function of *bd* oxidases. The properties of several mycobacterial cytochromes *bd* have been investigated^12,18,24,25^, but given they share only a low sequence identity (the lowest similarity score is 20%) with the two previously reported cytochrome *bd* structures it is difficult to rationalize the function of these cytochromes *bd*^18^. In the present study, we have determined a 2.8 Å cryo-electron microscopy (cryo-EM) structure of a dimeric *bd* oxidase from *Mycobacterium smegmatis* (*Msm*). In so doing, we have identified two potential oxygen access channels, which could be excellent targets for anti-tuberculosis drug discovery.

## Results and discussion

### Overall structure of *Mycobacterium smegmatis bd* oxidase

**Fig. 1:**
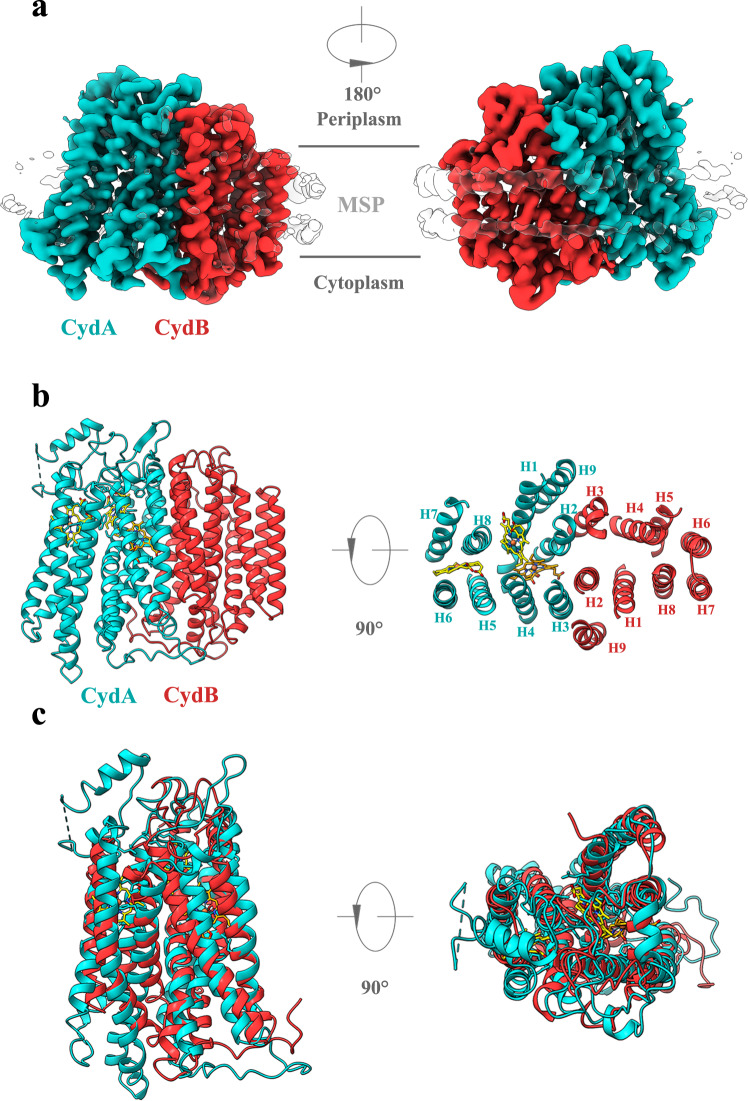
Overall structure of the *Msm bd* oxidase. **a** The *bd* oxidase cryo-EM density map at 2.8 Å resolution. MSP, membrane scaffold protein. **b** Cartoon representation of the *bd* oxidase consisting of subunits CydA (red) and CydB (cyan). **c** Structural superposition of CydA and CydB.

*Msm bd* oxidase was recombinantly expressed and purified to homogeneity (Supplementary Fig. 1a–d). The purified *Msm bd* enzyme is a stable and functional assembly with a turnover number of 21.6 ± 2.8 e^−^ s^−1^ (Supplementary Fig. 1e). A 2.8 Å resolution structure was determined in lipid nanodiscs using cryo-EM. Details for data collection and model statistics are provided (Supplementary Fig. 2 and Supplementary Table 1). Although the construct design, expression, and purification of *Msm bd* oxidase were performed according to a procedure described previously for *G. th*^19^ and *E. coli*^20,21^ *bd* oxidases, only CydA and CydB were observed. No additional small subunit similar to CydS/X or CydH/Y found in the *G. th*^19^ and *E. coli*^20,21^ *bd* oxidases were observed in the *Msm* complex, noting that in the *G. th* and *E. coli* studies, the genes coding for the associated subunits CydS/X of *G. th*^19^ *bd* oxidase and CydH/Y of *E. coli*^20,21^ *bd* oxidase were not included in the corresponding expression plasmid. Nonetheless, these two small subunits were observed to co-elute upon purification in both complexes. Thus, we believe that the *Msm bd* oxidase contains only two core subunits, CydA and CydB (Fig. 1a, b, and Supplementary Figs. 3, 4). This hypothesis is further supported by a BLAST search of the mycobacterial genomes which did not identify any subunits homologous to CydS/X and CydH/Y (Supplementary Fig. 5). In addition, *Msm bd* oxidase in the absence of associated subunits is active, whereas the *E. coli bd* oxidase is not active in the absence of its associated subunits^26^. In summary, mycobacterial *bd* oxidase appears to only consist of the two core subunits and no other units. Notably, there is a high structural similarity between the *G. th* and *E. coli* counterparts compared to *Msm* core subunits, except for a little difference in the topology of CydB subunits between *Msm* and *E. coli* (Supplementary Fig. 6).

In terms of secondary structure, *Msm bd* oxidase, CydA, and CydB possess a common fold each consisting of nine transmembrane helices (TMHs). These two subunits are related by an approximate two-fold rotational axis of symmetry. The TMH domains can be divided into two four-helix bundles and one additional peripheral helix (Fig. 1c). The domains of the two subunits superpose well with a root mean square deviation of 3.6 Å for all the Ca atoms (Fig. 1c) and thus suggest that CydA and CydB evolved by a gene duplication event. The three heme groups (*b*_558_, *b*_595_, and *d*) are clearly visible in the CydA subunit (Fig. 1b and Supplementary Fig. 3). Given the two other *bd* oxidases have associated subunits in their structures, our structure suggests that their presence is species-dependent.

### Q-loop of *M. smegmatis bd* oxidase

The *bd* oxidases are divided into the S (short)- and the L (long)-subfamilies^4^. This is according to the length of the region of polypeptide referred to as the Q-loop (quinol-binding domain). Mycobacterial cytochromes *bd* belong to the S-subfamily^20^. The Q-loop of CydA has a water-exposed domain (segment 257–339), connecting the 6 and 7 in the hydrophilic extracellular space^19–21^. It has two parts, one associated with the N-terminal domain (Q_N_) and the other with the C-terminal domain (Q_C_). The Q_N_-loop plays a functional role in the binding and oxidation of the quinol^27,28^ and the Q_C_-loop is needed for the assembly/stability of the enzyme^22,23,25^.

**Fig. 2:**
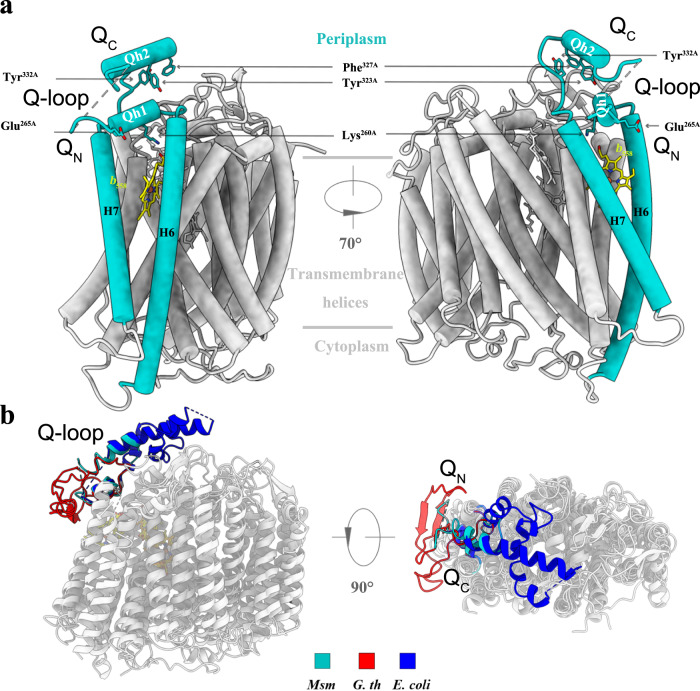
The Q-loop in *bd* oxidases. In CydA, a hydrophilic region between the transmembrane helices 6 and 7 harbors the quinol-binding site and has thus been named the Q-loop. **a** The N-terminal and C-terminal Q loops are rigid and well-ordered helical segments, but the linking region between them is not resolved in the maps. The residues labeled are important for stability or activity. **b** The *bd* oxidases from *G. th*, *E. coli*, and *Msm* are superimposed. The Q-loop domains from *G. th*, *E. coli*, and *Msm* are shown in red, blue, and cyan, respectively.

In the present study, the densities corresponding to the Q-loop without and with aurachin D (a quinone analog inhibiting mycobacterial cytochrome *bd*) bound are not completely resolved (Supplementary Figs. 2 and 7). A comparison of these two structures does not show any conformational changes which may be a function of the resolution of the data or the fact that the region around the aurachin D is inherently disordered. Aurachin D is bound to the Q_N_-loop^20^, potentially stabilizing the Q-loop, and has the ability to inhibit the activity of *Msm* cytochrome *bd*^29^. The structure of the Q_N_-loop in the *E. coli* enzyme is also not fully resolved^20,21^. Therefore, these structural data suggest that the Q_N_-loop is intrinsically flexible. Its flexibility may be required for the rapid binding and release of the quinols. The remaining segments of the Q-loop in the *Msm bd* oxidase structure are well resolved. At the periplasmic side of TMH 6, there is a short horizontal helix, Q*h1*, that includes the highly conserved residues Lys^260^ and Glu^265^ (Lys^252^ and Glu^257^ in *G. th* and *E. coli*)^19–21^, critical for quinol substrate binding and electron transfer^28^ (Fig. 2a). The structure of *Qh1* is conserved with respect to the *G. th* and *E. coli* enzymes^19–21^ (Fig. 2b). It is noteworthy that the Q_C_-loop here adopts a rigid secondary structure with a horizontal Q*h2* (residues 317–327), that emerges from the flexible Q_N_-loop part and covers the periplasmic surface of CydA. Site-directed mutagenesis and whole-bacteria assays, performed on the *Mycobacterium tuberculosis* (*Mtb*) *bd* oxidase, demonstrated that the regions corresponding to the Q*h2* and the equivalent residues Tyr^323^, Phe^327,^ and Tyr^332^ nearby the Q*h2* stretch are essential for the function of the oxidase^25^ (Supplementary Fig. 8). In *Msm bd* oxidase, we observe that these three residues are involved in the interactions with Q*h1* and the periplasmic loop between TMH 8/9, located at the periplasmic surface of heme groups *b*_558_ and *b*_595_, and as a result potentially affect the cofactor and quinol binding (Fig. 2a). Tyr^323^ forms the van der Waals interaction with Pro^258^ in the *Qh1* region, and Phe^327^ and Tyr^332^ form the stacking interactions with Trp^404^ in the loop TM8/9 region. So the observed structural features are in agreement with the previous functional characterization of the *Mtb* enzyme^25^. Additionally, according to the structural superposition between *Msm* and *E. coli* CydA subunits (Supplementary Fig. 6), the corresponding residues of Tyr^323^, Phe^327^, and Tyr^332^ in *Msm* are His^314^, Tyr^353^, Ser^379^ in *E. coli*, respectively. His^314^ and Tyr^353^ also form van der Waals interactions and a stacking interaction, respectively, with Thr^251^ in the *Qh1* region and Trp^451^ in the loop TM8/9 region. Hence, these interactions in the *Msm bd* oxidase are very similar to those observed in the *E. coli* complex. It is also worth noting that the folding of the Q_C_-loop region is different (Fig. 2b), compared to the other *bd* oxidases, though the Q-loop domain of *Msm bd* oxidase should be remembered these belong to the short Q-loop class (Supplementary Fig. 9)^20^. This array of differences suggests that the Q-loop domain could be used as a marker for evolutionary analysis of *bd* oxidases in prokaryotes (Supplementary Fig. 10).

### Electron transfer in *Msm bd* oxidase

**Fig. 3:**
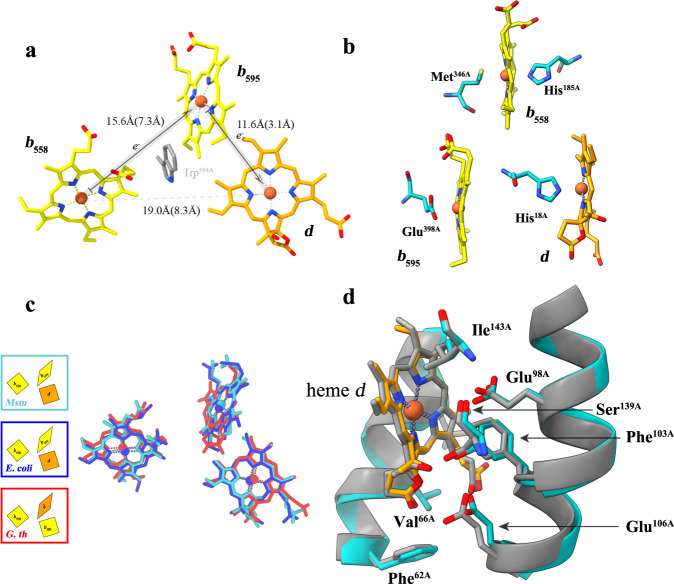
Cofactor organization in *Msm* cytochrome *bd*. **a** Triangular arrangement of the heme cofactors in CydA. Heme edge-to-edge distances are indicated by numbers in the parentheses. **b** Axial amino acid ligands of the heme cofactors. **c** Heme superposition between *E. coli*, *G. th*, and *Msm*. **d** The superimposed heme *d* sites from *Msm* and *E. coli*.

The three heme groups (*b*_558_, *b*_595_, and *d*) unambiguously identified in *Msm bd* oxidase are organized in a triangulated arrangement near the periplasmic side of CydA (Fig. 3a). In this structure, the low-spin *b*_558_ is within the transmembrane core of subunit CydA, adjacent to the Q_N_-loop segment. Its axial ligands are conserved residues His^185^ and Met^346^ (His^186^ and Met^325^ in *G. th*; His^186^ and Met^393^ in *E. coli*)^19–21^ (Fig. 3b). Heme *b*_595_ is located closer to the periplasmic side and is ligated by Glu^398^ (Glu^378^ in *G. th*; Glu^445^ in *E. coli*)^19–21^ (Fig. 3b). There is a conserved Trp^394A^ between heme groups *b*_595_ and *b*_558_ that may mediate electron transfer^19–21,30^. The third cofactor heme *d*, the site of oxygen binding and reduction, is positioned at the center of CydA and the invariant His^18^ (His^21^ in *G. th*; His^19^ in *E. coli*) appears to be ligated to the heme iron on one side^19–21^.

Although the *bd* oxidases of *G. th* and *Msm* are from the same S-subfamily, the relative arrangement of the three heme groups are strikingly different^19^ (Fig. 3c). The organization of the redox center in *Msm* cytochrome *bd* is similar to that reported for the L-subfamily as exemplified by *E. coli bd* oxidase^20,21^. A structural superimposition (Fig. 3c) shows the two heme groups are located in the same position in the CydA, the distances between the central iron atoms are consistent as the orientations of the heme planes relative to the membrane plane. Therefore, the cofactor location is a conserved feature of the three respiratory *bd* oxidases. On the opposite side of the heme *d*, Glu^98^ (Glu^101^ in *G. th*; Glu^99^ in *E. coli*) acts as the axial ligand. Glu^98^ here is 6.2 Å from the central iron atom. The equivalent distances in *G. th* and *E. coli* are 2.1 and 6.0 Å, respectively^19–21^. This voluminous cavity is suggested to be used for the binding of substrates such as oxygen, roofed by the hydrophobic Ile^143^ and Phe^103^ (Ile^144^ and Phe^104^ in *E.coli*)^20,21^. The location and surrounding environment of the cofactors in the *bd* oxidase are highly conserved between *Msm* and *E. coli* (Fig. 3d). Collectively, it is suggested that a sequential electron transfer from heme *b*_558_ via heme *b*_595_ to heme *d* also exists in the mycobacterial *bd* oxidases^20,21^.

### Two oxygen access channels in *Msm bd* oxidase

**Fig. 4:**
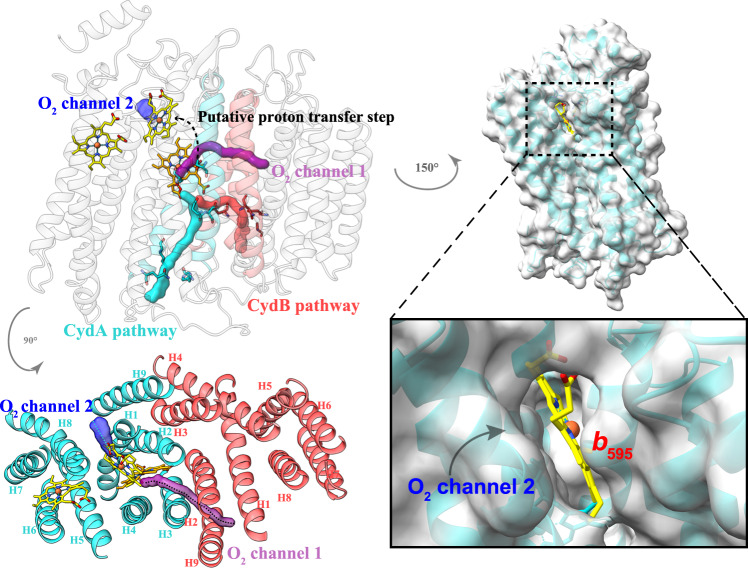
Proton/gaseous substrate channels in the *Msm bd* oxidase. Subunits CydA/B are shown in cartoon representation. The putative dioxygen and proton channels are labeled. The heme groups are shown as stick models. The proton transfer step from heme *b*_595_ to heme *d* is identified by a black dashed path.

Cytochromes *bd* play a role in energy metabolism with high O_2_ affinity under hypoxic conditions^4,12^, conditions often encountered by the microorganisms in their natural habitats. The dioxygen has to bind to heme *d* and then is reduced at this position^19–21^. In *E. coli bd* oxidase, the O_2_-channel acts as a pathway for direct oxygen diffusion from the membrane interior to the heme *d* reaction site. It is formed by a small direct hydrophobic channel, which starts above Trp^63^ (Trp^67^ in *Msm*) at the membrane interface between TMH1 and TMH9 of CydB and extends further to heme *d* on CydA^20,21^. The corresponding residue Trp has been demonstrated to be essential for *bd* activity in *Mtb*^25^. Noteworthy, in the *Msm* structure, the channel is also formed by a conserved structural topology and residues according to a comparison between the *bd* oxidases from *E. coli* and *Msm* (Supplementary Fig. 11), which suggests that the oxygen here may also access the active site through this conserved O_2_-channel (identified as channel 1) (Fig. 4). Intriguingly, there is an additional accessible channel directly connecting to the protein surface and extending to the heme *b*_595_, which is also identified in the *G. th* enzyme^19^. This channel has also been proposed for the oxygen entry site in *G. the bd* oxidase^19,20^, which is blocked by the single-transmembrane subunit CydH in the *E. coli* enzyme^20,21^. In addition, the heme *d* in this structure is buried deeper inside the subunit CydA and the penetration of dioxygen from this cavity into heme *d* is also blocked by heme *d* itself^21^. However, the channel is accessible in the *Msm* enzyme and previous studies have reported that the high-spin heme *b*_595_ could be the second reaction site for O_2_^5,31^_._ Therefore, this channel (channel 2) is very likely to be an alternative pathway to guide dioxygen to heme *b*_595_, which may further sustain energy metabolism in the bacterial cell and enhance mycobacterial survivability in the host. It has been reported that the threshold pO_2_ (O_2_ tension) of the growth medium for the induction of *cyd* gene cluster in *M. smegmatis* (ca. 1% air saturation)^32^ is significantly lower than that of *E. coli* (10% air saturation)^33^, suggesting that the *Msm bd* oxidase has a different functional or kinetic range with respect to oxygen availability compared to that of *E. coli*^32^. Overall, therefore, the two oxygen channels in *Msm bd* oxidase are reasonably proposed based on these structural features and the previously described studies. Future investigations are needed to determine whether these two catalytic reactions take place at the same time.

Although the *bd* oxidases do not pump protons from the cytoplasmic side to periplasmic side, producing the proton motive force across the membrane, the pathway for proton uptake from the cytoplasm is crucial to reduce dioxygen to water^4^. Two proton pathways in subunits CydA and CydB from the cytoplasmic side to the active site have been proposed in *G. th*^19^ and *E. coli* enzymes^20,21^. These studies indicate two hydrophilic channels for proton transfer. Based on the superimposition and structural analysis between the *bd* oxidases from *G. th* and *E. coli*^19,20^, the relatively conserved hydrophilic residues in our model, along the canonical CydA and CydB pathways, are His^125.A^, Gln^36.A^, Glu^106.A^, Ser^107.A^, Ser^139.A^ to Glu^98.A^, and Asp^25.B^, Asp^62.B^, and Asn^64.B^ (Fig. 4, Supplementary Fig. 11). Given the conserved identity of the proton pathways in mycobacterial enzymes, they are also likely to facilitate proton transfer for dioxygen reduction at the heme *d* site. In addition, in terms of the dioxygen reduction at the heme *b*_595_ site, there must be an additional proton transfer step from heme *d* to heme *b*_595_ in order to deliver protons to the oxygen reduction site (Fig. 4), which may be potentially similar to that of the *G. th* enzyme^19^. According to the current structure, the heme propionate of heme *d* is in a hydrophobic environment without any charge compensation. It is thus very likely protonated and supplies protons for unresolved water molecules here that connect heme *b*_595_ to heme *d*. These protons would be replenished via the CydA/B proton pathways.

**Fig. 5:**
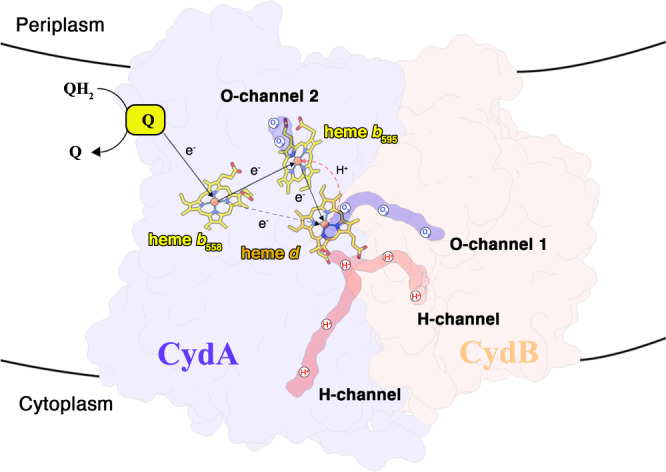
A schematic diagram showing the electron/proton transfer pathway in *Msm bd* oxidase and the relevant oxygen entry pathway. The heme groups are shown as in stick models. The putative dioxygen and proton channels are labeled. Electron transfer directions are shown in black arrows.

Overall, the electron released from the quinol bound at the quinone-binding site is transferred, in turn, to the prosthetic groups heme *b*_585,_ heme *b*_595_ to heme *d*. At the same time, the oxygen molecule that is diffused to the heme *b*_595_ and/or heme *d* sites is reduced to water, a process that is involved in conducting protons to the oxygen-binding site through the CydA/B pathways (Fig. 5).

Cytochromes *bd* is ubiquitous among prokaryotes (but not present in eukaryotes) and is now attracting attention as promising targets for next-generation antibacterials. Here, we have determined the 2.8 Å cryo-EM structure of *Msm bd* oxidase. The overall fold is similar to the two other previously reported *bd* oxidases but exhibits several different features, including the fold of the Q-loop and the number of associated subunits. In addition, we have identified two potential oxygen access channels that look to be also present in *G. th* and *E. coli* cytochromes *bd*. The quinol-binding site located in the Q-loop has been proposed to be a target for drug discovery. However, the structure of the Q-loop has not been fully determined, thus posing a challenge for the design of quinol-type inhibitors. The two oxygen-conducting O_2_-channels could be alternative targets for the discovery of anti-tuberculosis drugs.

## Methods

### Bacteria strain and culture

The *cydAB* gene was cloned into pMV261 plasmid with a 10x His tag at the C-terminus of *cydB*. The primers are listed in Supplementary Table 2. Expression was achieved by electroporation of the plasmid into strain *Msm* mc^2^ 51^34^. A volume of 1 mL strain stock was added to 24 mL LB broth (0.1% Tw80, 50 μg/mL kanamycin, and 20 μg/mL carbenicillin) and cultured overnight at 37 °C and 220 rpm. Next, 4 mL pre-culture aliquots were transferred to 1 L LB broth (rubber plug, 0.1% Tw80, 50 μg/mL kanamycin, and 20 μg/mL carbenicillin) and cultured at 37 °C and 220 rpm. When the OD_600_ reached 0.8, 5 mL of 40% acetamide was added to induce the expression of the target protein over 3 days at 25 °C and 220 rpm.

### Protein purification and characterization

The purification procedure followed a previous study but with a few modifications^30^. Membranes of the cells were extracted in buffer (20 mM HEPES, pH 7.4, 100 mM NaCl), and then stirred slowly at 4 °C for 2 h with 1% (w/v) dodecyl-beta-d-maltoside (DDM). The supernatant after centrifugation was loaded onto a Ni-NTA column and the eluted fraction including the protein of interest was loaded onto a Superdex 200 (GE Healthcare) column equilibrated in a buffer containing 20 mM HEPES, pH 7.4, 100 mM NaCl, and 0.02% (w/v) DDM. The peak fractions were analyzed by SDS–PAGE (sodium dodecyl sulfate–polyacrylamide gel electrophoresis), then pooled and concentrated to 6 mg/mL.

### Preparation of reduced quinol substrate

2,3-Dimethyl-1,4-naphthoquinone (DMNQ, CAS 2197-57-1) was synthesized by WuXi AppTec. DMNQ reduction was performed following previously published protocols with some modifications^35^. To prepare the reduced quinol, DMNQH_2_, 20 mM DMNQ was ultrasonically dissolved in 1 mL ethanol with 6 mM HCl. A few grains of sodium borohydride (NaBH_4_) were then added to obtain a fully reduced, colorless solution in the ice-bath. An appropriate amount of HCl was used to quench the mixture under the protection of argon. The quinol solution was stored at −80 °C.

### Oxygen consumption assay

Oxidase activity was determined according to the previous studies^20,36,37^. Oxygen consumption was monitored with a Clark-type oxygen electrode (Hansatech Chlorolab 2) in the buffer 20 mM HEPES, pH 7.4, 100 mM NaCl, 0.04% DDM, and 10 mM DTT at room temperature. To begin the assay, 480 μL buffer was first added until the oxygen equilibrium. 20 μL DMNQH_2_ was then added and the substrate autoxidation rate was recorded. The reaction was started by the addition of 0.8 μM *bd* complex. The time course for oxygen consumption was curved with GraphPad prime 6.0 software, from which an estimate of the observed pseudo-first-order rate constant (*k*_obs_) is obtained (corrected for autoxidation). This assay included four groups of parallel experiments.

### Reconstruction of cytochrome bd into nanodiscs

MSP1D1 was used to reconstruct the nanodisc and the purification and reconstruction followed the reported study^38^. Briefly, cytochrome *bd*, MSPD1, and POPC were mixed with a stoichiometry of 1:4:160 and incubated at 4 °C for one hour. Next, 200 μL of resuspended Bio-Beads (0.5 g/mL) were added twice with an interval of 30 min to remove the detergents. After 12 h of incubation, the supernatant was applied to a Superdex 200 (GE Healthcare) column equilibrated in 20 mM HEPES, pH 7.4, 100 mM NaCl buffer. The peak fraction was collected and concentrated.

### Cryo-EM sample preparation and data collection

Aliquots (4 μL) of reconstructed nanodisc-cytochrome bd at a concentration of 1 mg/mL were applied to glow-discharged Quantifol Cu 1.2/1.3 (mesh 300) grids. For cytochrome bd and aurachin D complex, 0.35 mM aurachin D was added and incubated with nanodisc-cytochrome bd for half an hour before sample vitrification. Glow discharge was accomplished by adding an H_2_ and O_2_ mixture in the Gatan Solarus 950 for 25 s. After blotting for 3 s with a blot force of −2, grids were flash-frozen in liquid ethane cooled by liquid nitrogen using an FEI Vitrobot operated at 8 °C and 100% humidity. For cytochome bd complex without aurachin D, data collection was achieved using the Titan Krios electron microscopy operated at 300 kV with a Gatan K3 detector at a magnification of SA ×29,000. Images were recorded in super-resolution mode binned to a pixel size of 0.82 Å/pixel. Data acquisition was achieved by using serialEM^39^. Images were collected with 40 frames and a total dose of 60 e^−^/Å^2^. The defocus range was set to 1.2–1.8 μm. For cytochrome bd–aurachin D complex, images were recorded using an FEI Titan Krios electron microscopy operating at 300 kV with a Gatan K2 detector at a magnification of ×165,000 with an energy filter, corresponding to a pixel size of 0.82 Å/pixel. Images were collected with 40 frames and a total dose of 60 e^−^/Å^2^ with a defocus range between 1.2 and 1.8 μm.

### Image processing

Dose-fractioned images were motion-corrected and dose-weighted by MotionCor2 software^40^. CTF estimation was performed by cryoSPARC^41^. 1,578,284 particles were automatically picked and extracted with a box size of 256 pixels^41^. 2D classification and 3D classification and refinement were all performed in cryoSPARC^41^. 50,000 particles were used to generate three classes in ab-initio reconstruction. The classes were used as templates for heterogeneous refinement with all selected particles. After a few rounds of heterorefinement, 270,938 particles converged into one class with a 3.4 Å initial map. These particles were used to perform a homogeneous refinement and local refinement to obtain a final resolution of 2.79 Å.

### Model building and refinement

The final map was sharpened automatically using a *B*-factor of 121.3 Å^2^ in cryoSPARC. The atomic model was manually built in Coot^42^ (version 0.8.9.1) using the crystal structure of *G.th* cytochrome *bd* (PDB: 5doq)^19^ as a template. Real-space refinement and validation of the final model were performed in Phenix (version 1.14)^43^. The local resolution map was calculated with ResMap^44^. All reported resolutions were based on the gold-standard FSC 0.143 criteria^45^. FSC_work_ and FSC_test_ were conducted to check for over fitting^46^.

All figures were created using UCSF Chimera^47^ or PyMOL^48^.

### Reporting summary

Further information on research design is available in the Nature Research Reporting Summary linked to this article.

## Supplementary information

Supplementary Information

Reporting summary

## Data Availability

The accession numbers for the 3D cryo-EM density map of *Msm bd* oxidase without and with bound AD in present study are EMD-30582 and EMD-31302, respectively. The accession number for the coordinates for the *Msm bd* oxidase without bound AD in this study is PDB: 7D5I. Source data are provided with this paper.

## Source data

Source Data
