# Supplementary material for: Cryo-EM structure of mycobacterial cytochrome bd reveals two oxygen access channels
Source: Nat Commun. 2021 Jul 30;12:4621. doi: 10.1038/s41467-021-24924-w (PMC8324918; doi:10.1038/s41467-021-24924-w)
Supplement: Supplementary file 1 — Supplementary Information [file 41467_2021_24924_MOESM1_ESM.pdf]

Supplementary Information

Cryo-EM structure of mycobacterial cytochrome *bd* reveals two oxygen access channels

Weiwei Wang, Yan Gao et al.

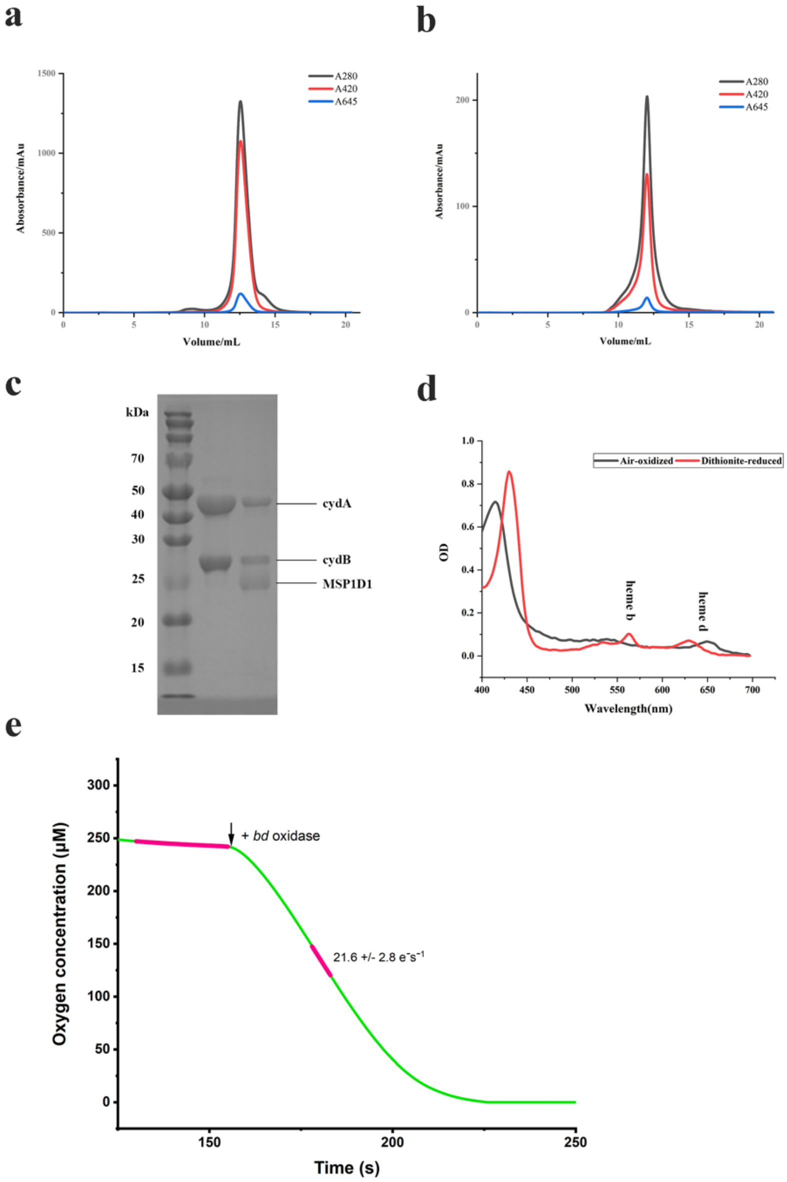

Supplementary Fig. 1 Purification and component identification of the *Msm* cytochrome *bd*. a The elution profile of the cytochrome *bd* from size exclusion chromatography (SEC). **b** The elution profile of the reconstructed nanodisc cytochrome *bd* from SEC. **c** SDS-PAGE of the pooled fraction from SEC in **a** and **b**. **d** UV-visible spectra of the dithionite-reduced (red) and air-oxidized (black) forms of the cytochrome *bd*. In the dithionite reduced spectrum, the absorbance peaks of the heme *b* (563 nm) and heme *d* (630 nm) are observed. **e** Oxygen reductase activity of *bd* oxidase samples is indicated. The purple trace before and after addition of enzyme represents the slopes used for turnover calculations.

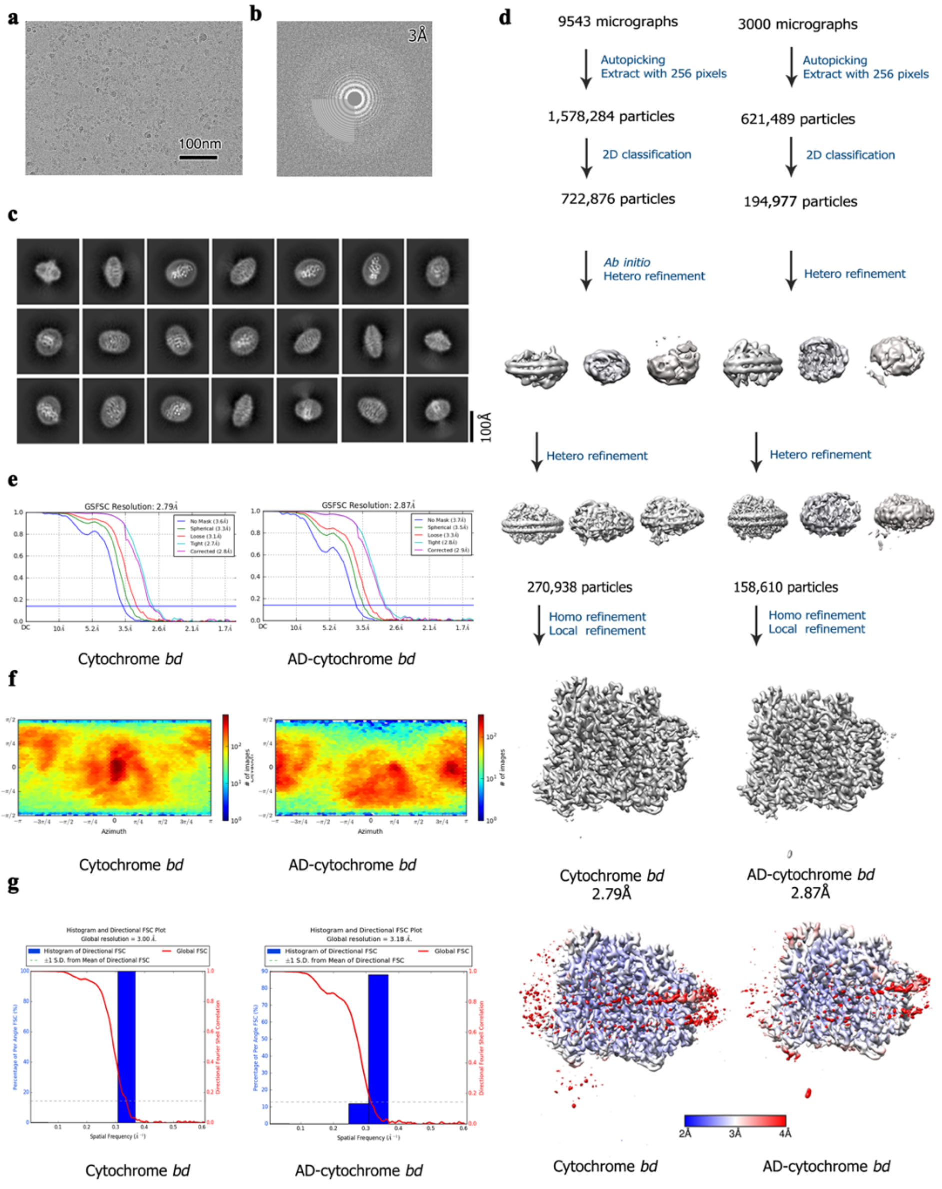

Supplementary Fig. 2 Cryo-EM data processing of *Msm* cytochrome *bd* complex. a Representative electron micrograph of 9543 micrographs obtained for the cryo-EM sample of cytochrome *bd*. Scale bar = 100nm. **b** Representative CTF fit of 9543 motion-corrected micrographs. **c** Representative 2D classification averages calculated from the finally selected 270,938 particles for cytochrome *bd*. **d** Data processing of cytochrome *bd* with and without aurachin D. **e** FSC curves of 3D reconstructions. **f** Viewing direction of all particles used in the final 3D reconstruction. **g** 3DFSC histogram of final map.

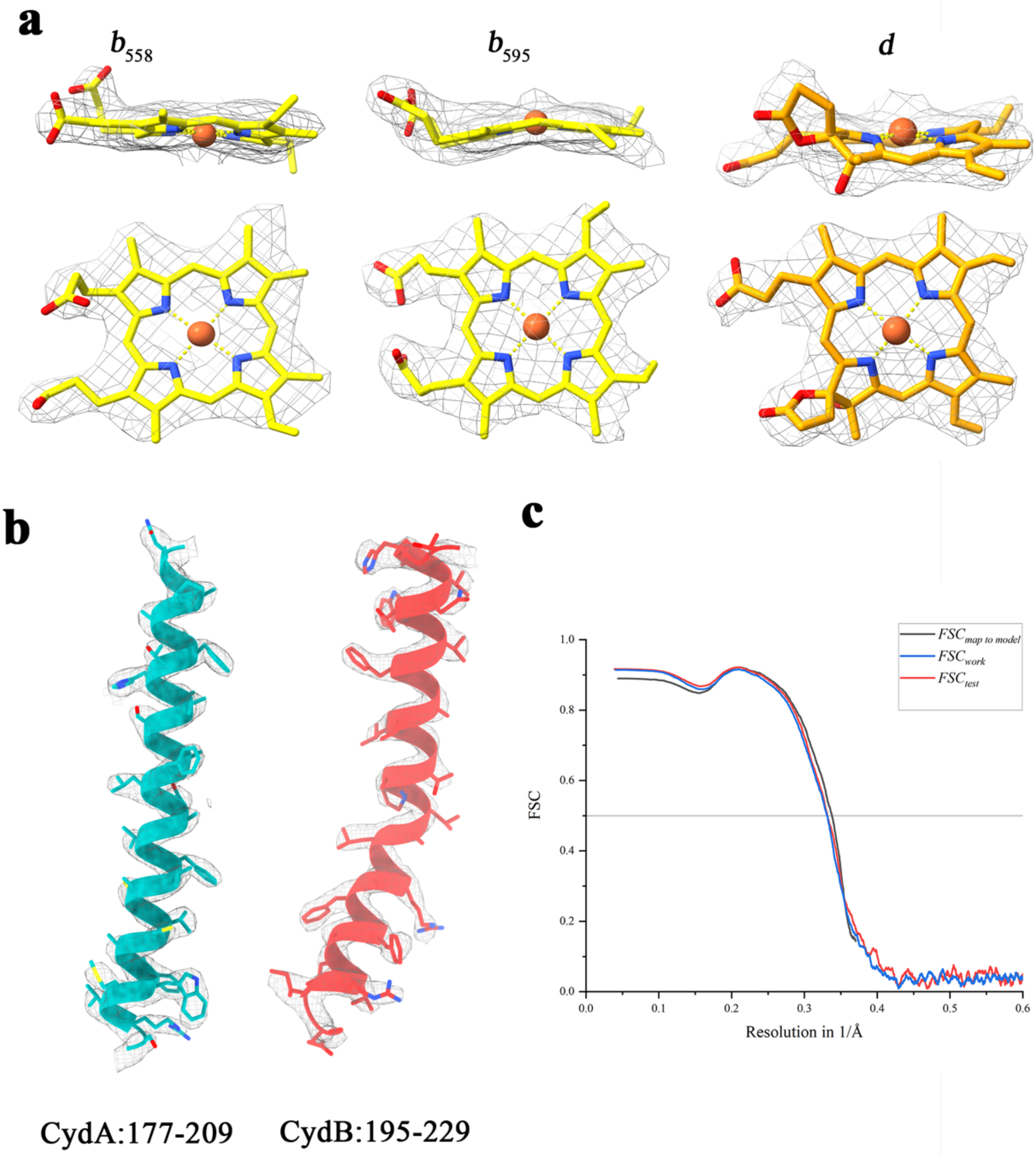

Supplementary Fig. 3 Cryo-EM map quality assessment and ligand representation for *Msm* cytochrome *bd*. Representative cryo-EM densities of (**a**) prosthetic groups, (**b**) helices**. c** FSC_{work} and FSC_{test} calculations.

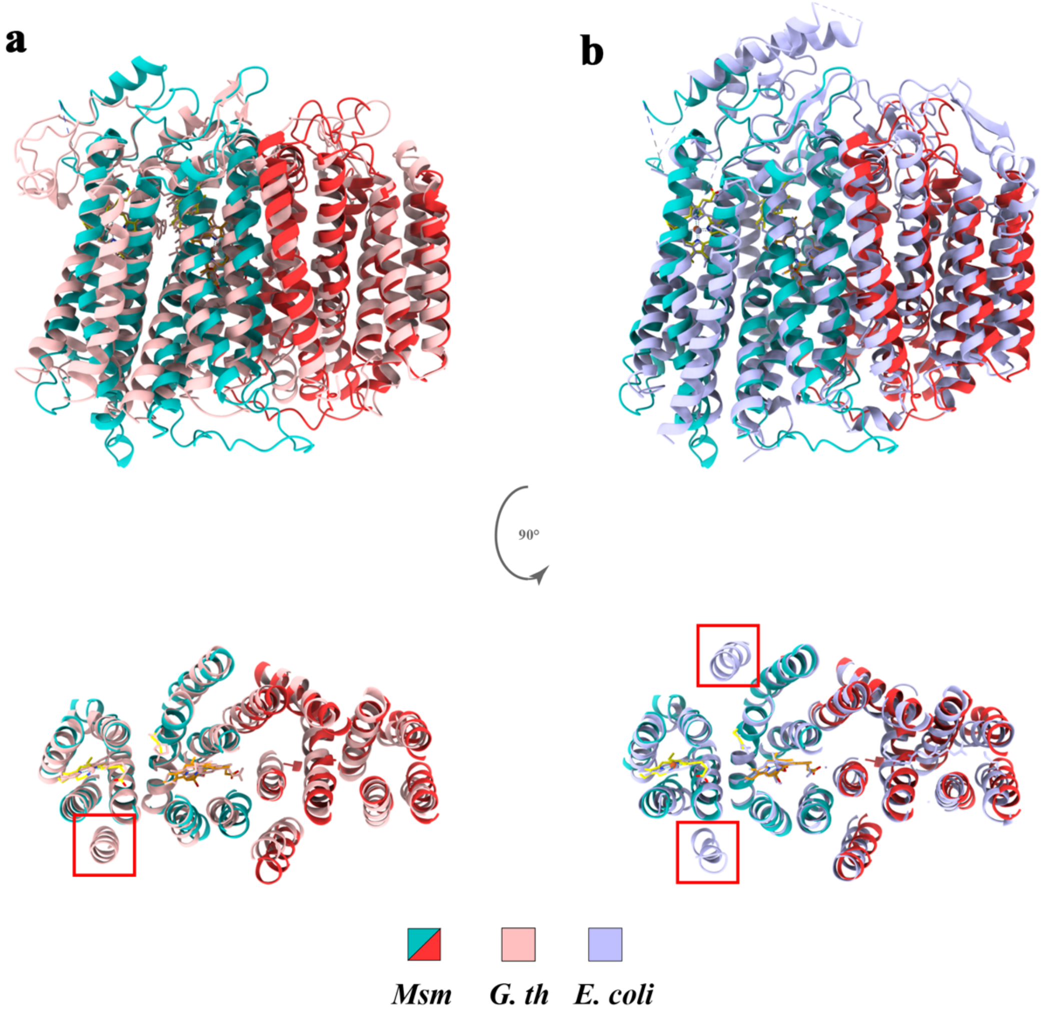

Supplementary Fig. 4 Structural alignment of cytochrome *bd* oxidases. Structural comparison of (**a**) *Msm* and *G. th* cytochrome *bd* oxidases, (**b**) *Msm* and *E. coli*. The overall arrangements of helixes are similiar in the three cytochrome *bd* oxidases. However, as shown in red squares, the subunits CydS in *G.th* (CydX in *E. coli*) and CydH in *E. coli* are missing in *Msm bd* oxidase.

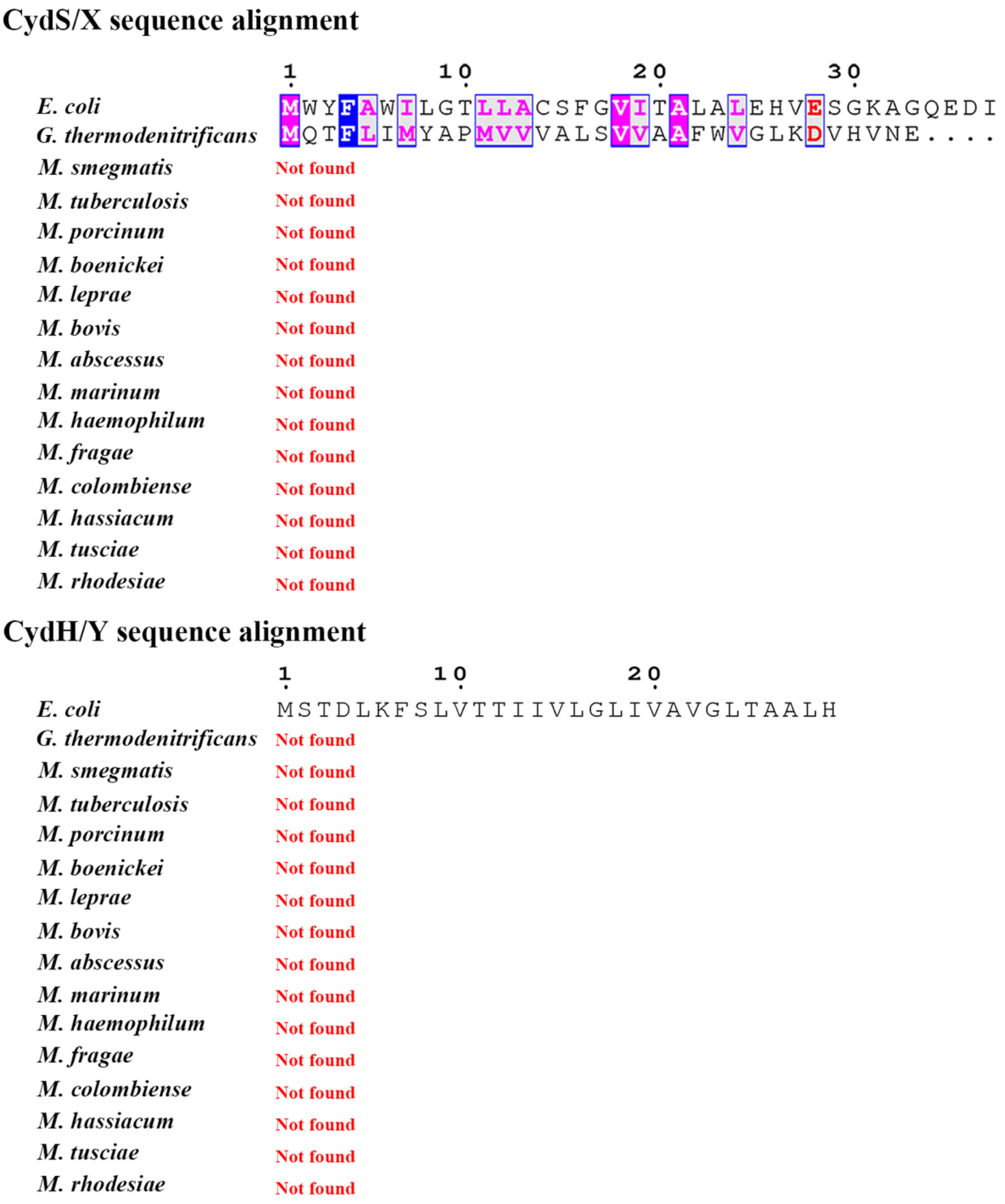

Supplementary Fig. 5 Sequence alignment of CydS/X and CydH/Y subunits. In order to evaluate the conservation of the subunits CydS/X and CydH/Y in the mycobacterial genomes, homologous sequences were detected through the NCBI BLAST. No homologous sequence of the CydS/X or CydH/Y was found in mycobacterial genomes. Some representative mycobacterial species are shown.

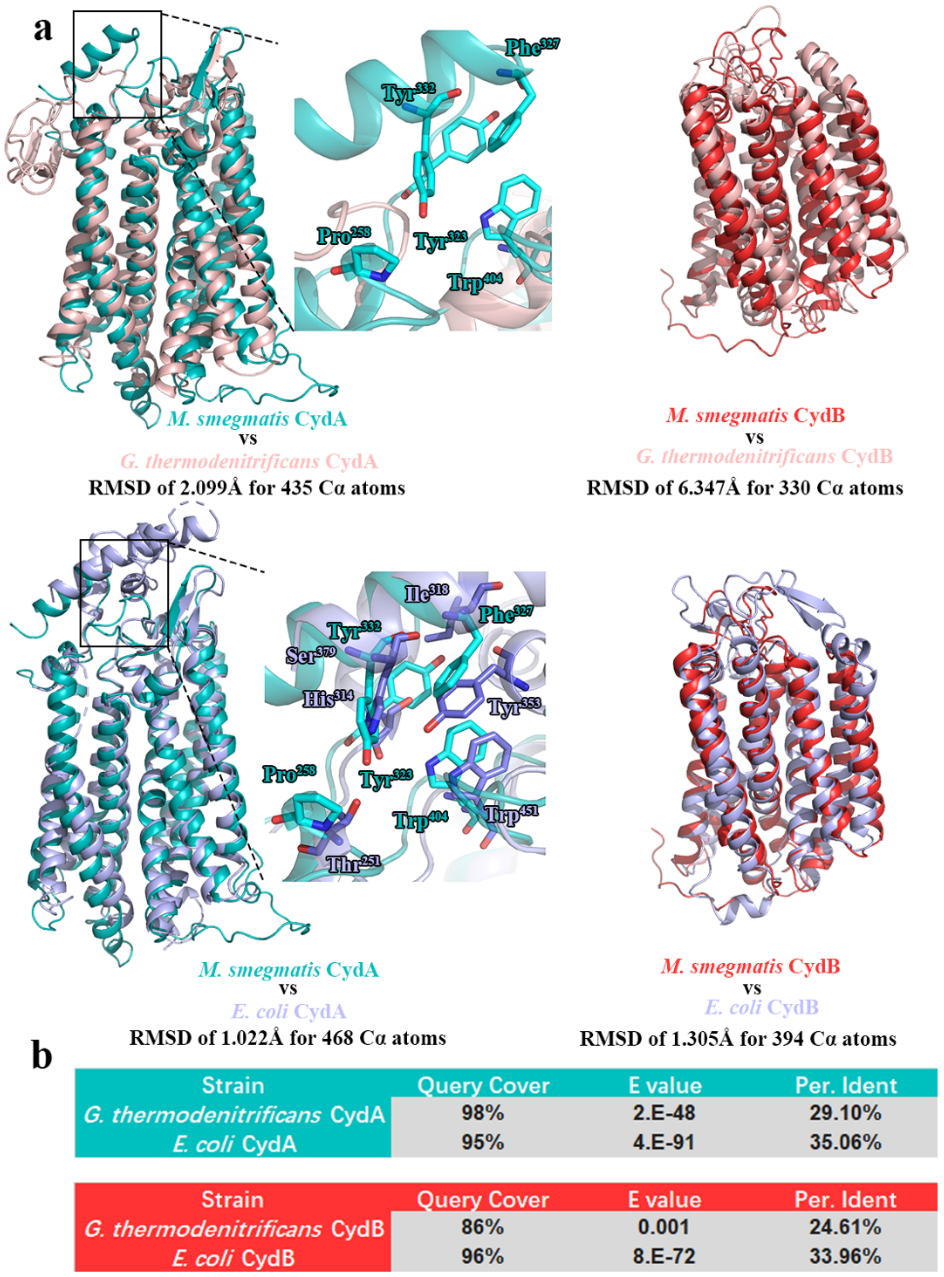

b

Strain	Query Cover	E value	Per. Ident
G. thermodenitrificans CydA	98%	2.E-48	29.10%
E. coli CydA	95%	4.E-91	35.06%

Strain	Query Cover	E value	Per. Ident
G. thermodenitrificans CydB	86%	0.001	24.61%
E. coli CydB	96%	8.E-72	33.96%

Supplementary Fig. 6 Subunits CydA and CydB of *Msm bd* and comparison with that of *G. th* and *E. coli*. a Structural superimposition of *Msm* CydA/B with *G. th* CydA/B (PDB code 5DOQ) and *E. coli* CydA/B (PDB code 6RKO), respectively. Three residues Tyr^{323}, Phe^{327} and Tyr^{332} of *Msm Qh2* region and the counterparts of *G. th* and *E. coli* are shown. RMSD, root mean square deviation. The subunits are colored accordingly. **b** Sequence alignment of *Msm* CydA/B with *G. th* CydA/B and *E. coli* CydA/B, respectively.

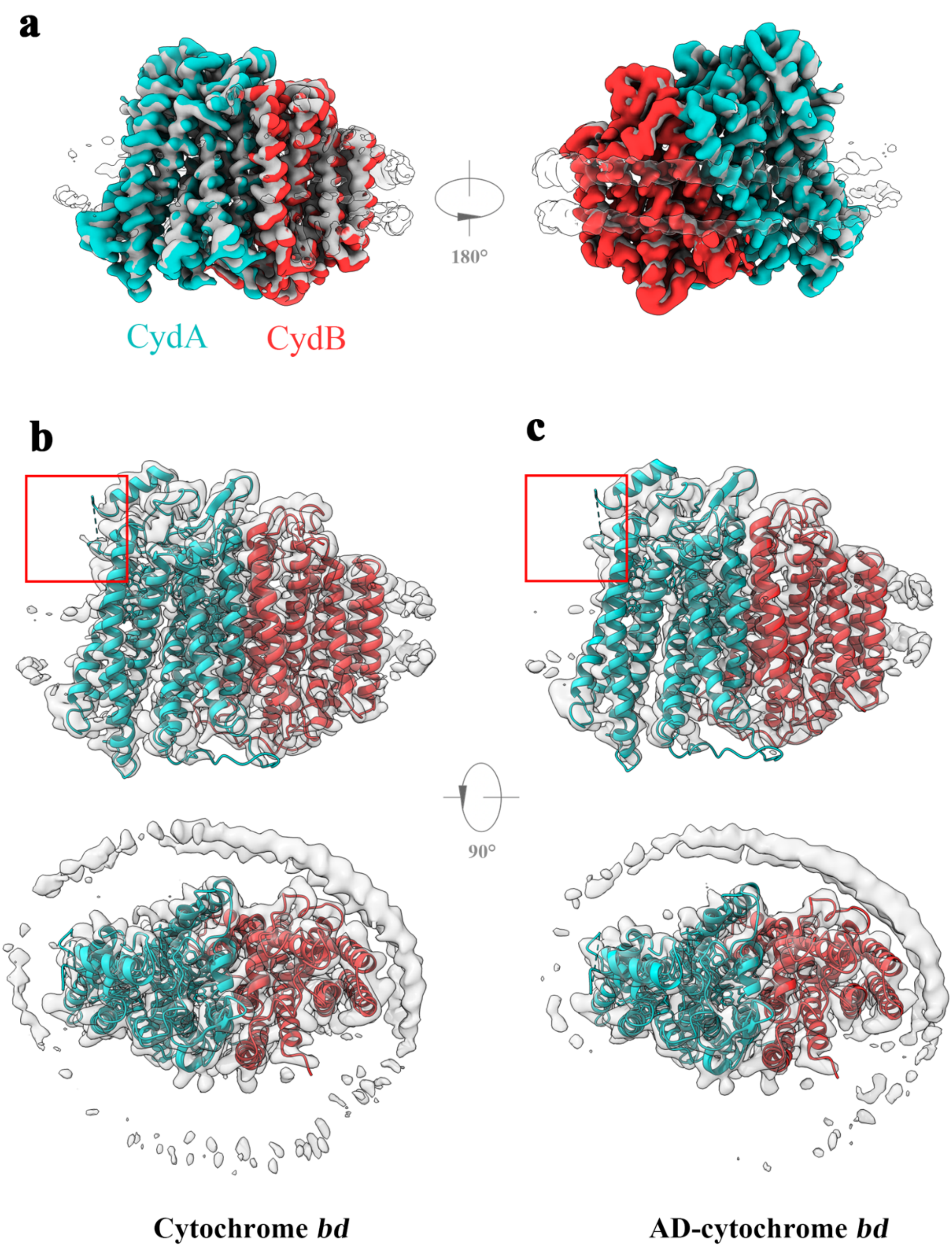

Supplementary Fig. 7 Cryo-EM map alignment and overall density of *Msm* cytochrome *bd* with and without aurachin D. a Alignment between the maps with and without aurachin D. **b** Front and top views of the *bd* oxidase map without aurachin D. **c** Front and top views of the *bd* oxidase density map with aurachin D. The densities corresponding to the Q-loop are noisy in both maps. Contour level is set to 0.3.

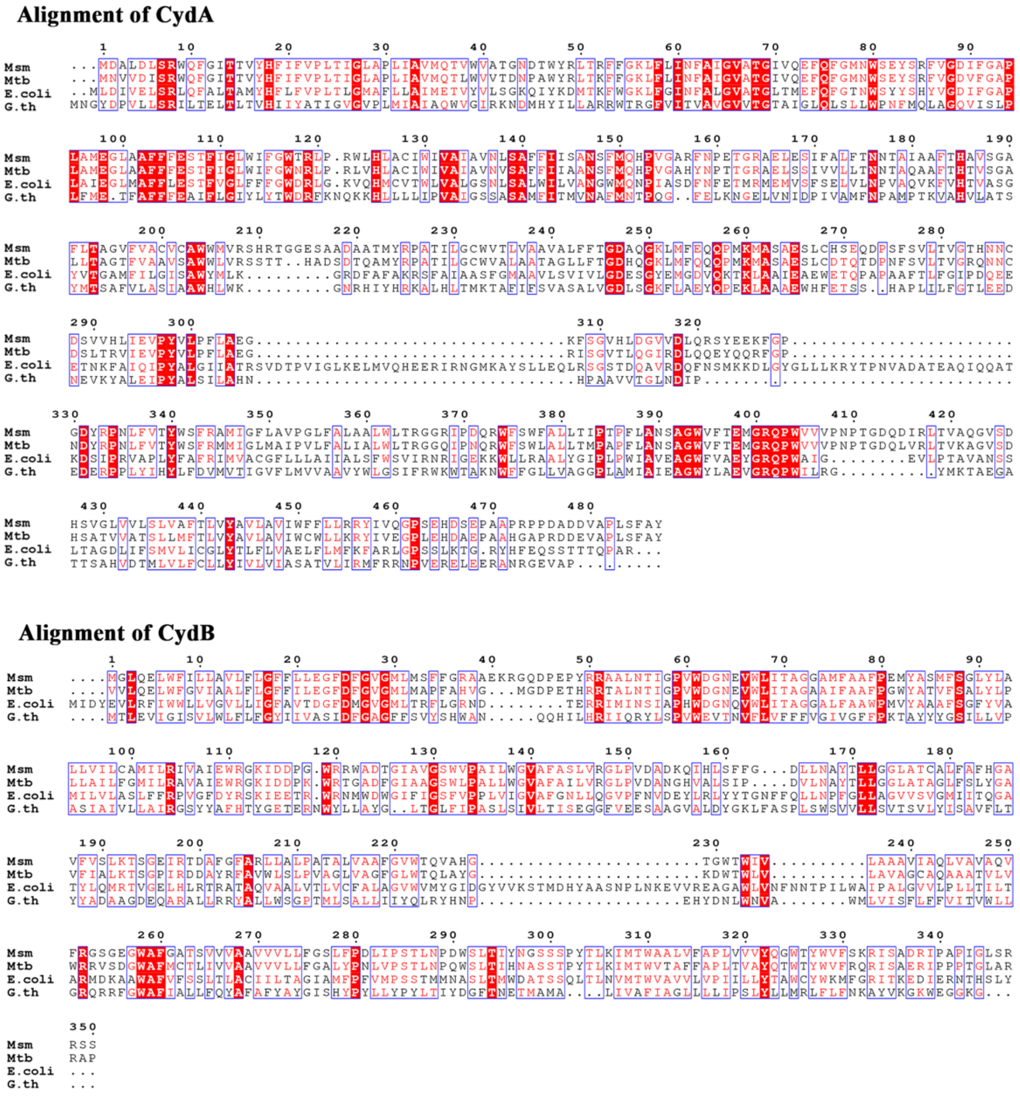

Supplementary Fig. 8 Sequence comparison for the *bd* oxidases.

Sequence alignment of CydA and CydB from *Msm, Mtb, E. coli* and *G. th.*

Alignment of Q-loop

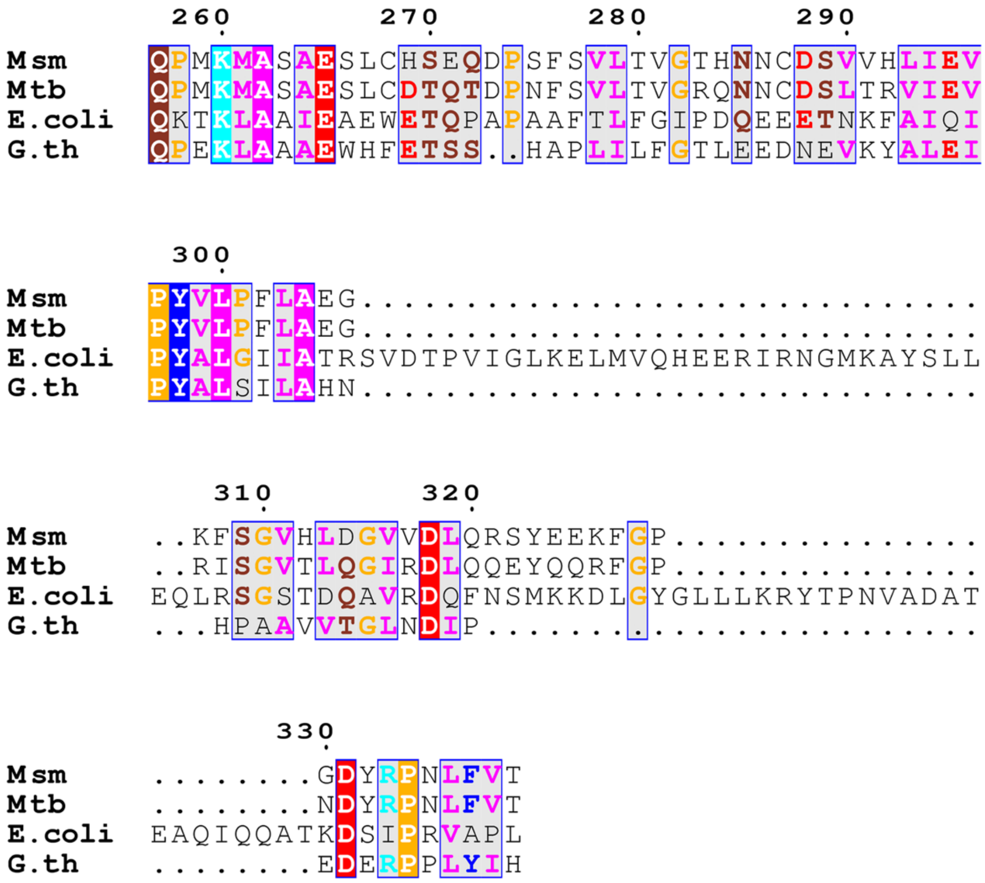

Supplementary Fig. 9 Sequence comparison of the Q-loop region in the *bd* oxidases. Lys^{260} and Glu^{265} in *Msm* and Lys^{258} and Glu^{263} in *Mtb* (Lys^{252} and Glu^{257} in *G. th* and *E. coli*) are conserved and critical for quinol binding and electron transfer.

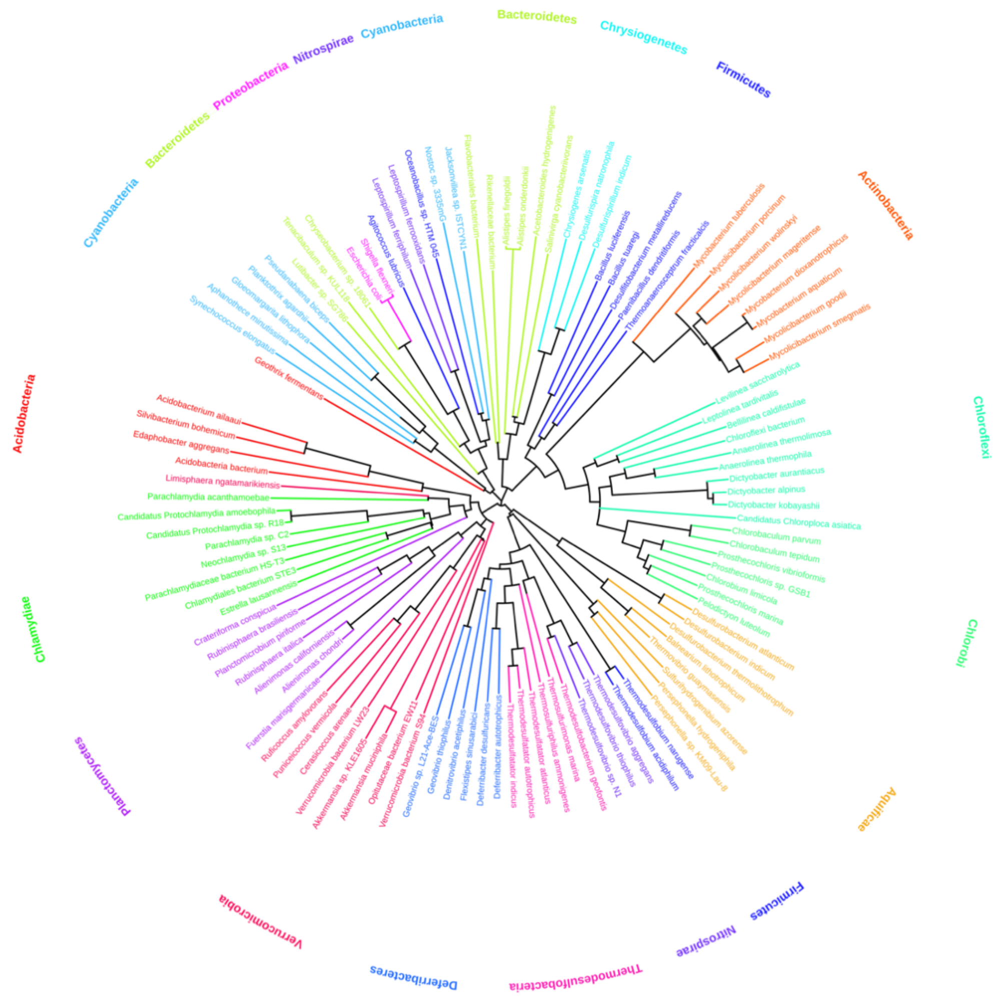

Supplementary Fig. 10 A circular phylogenetic tree showing the relationships between 120 Q-loop domain sequences of cytochrome *bd* oxidases based on Minimum Evolution (ME) method. The cytochrome *bd* oxidases from 18 bacterial phyla (Acidobacteria, Actinobacteria, Aquificae, Bacteroidetes, Chlamydiae, Caldithrix, Chlorobi, Chloroflexi, Chrysiogenetes, Cyanobacteria, Deferribacteres, Firmicutes, Nitrospirae, Planctomycetes, Proteobacteria, Thermi, Thermodesulfobacteria and Verrucomicrobia) are clustered and classified into corresponding groups according to the evolutional relationships of their Q-loop regions. The image was generated by MEGA[1] and further improved by iTOL[2].

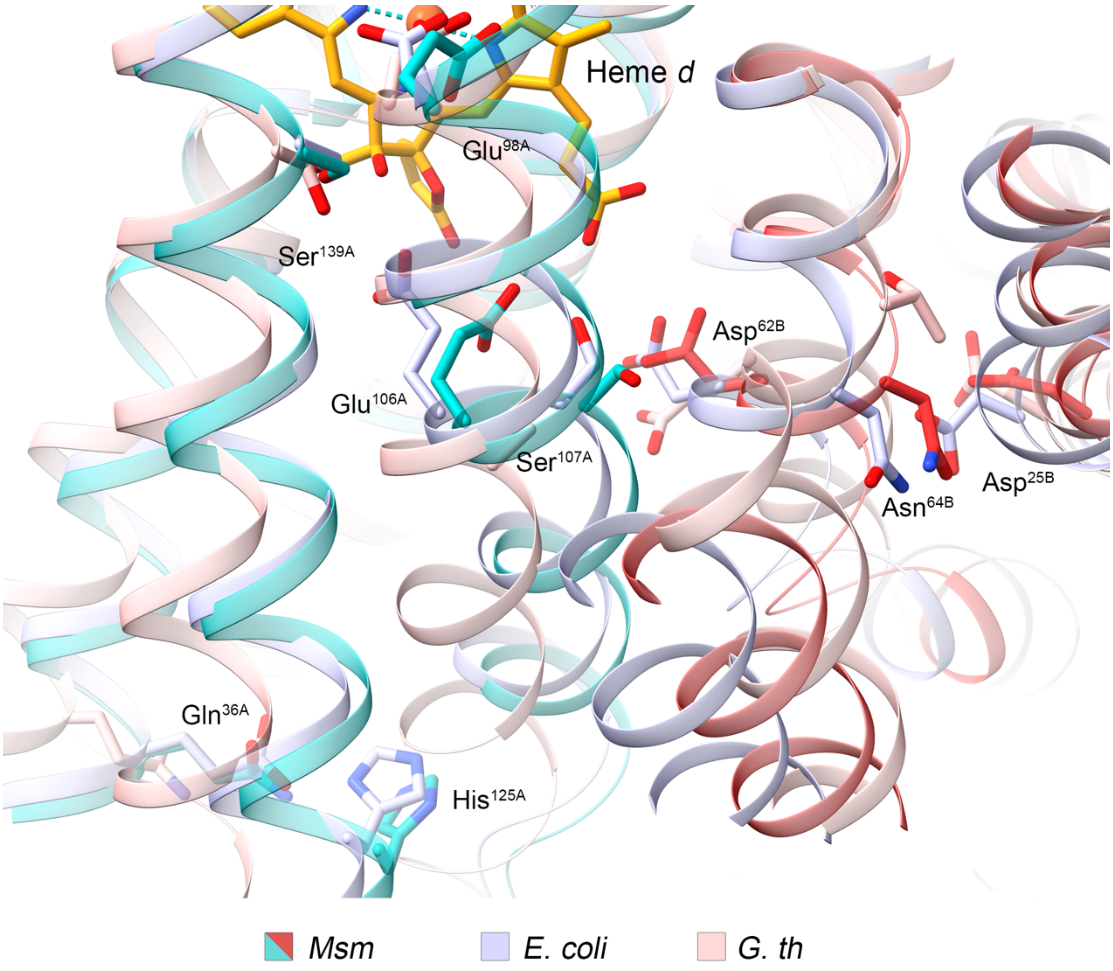

Supplementary Fig. 11 Proton transfer pathway in three different cytochrome *bd* oxidases. Hydrophilic residues in *Msm* CydA (His$^{125.A}$, Gln$^{36.A}$, Glu$^{106.A}$, Ser$^{107.A}$, Ser$^{139.A}$, Glu$^{98.A}$) and CydB (Asp$^{25.B}$, Asn$^{64.B}$, Asp$^{62.B}$) are relatively conserved. Subunits are colored in cyan and red, respectively. Protons are conducted by these hydrophilic residues through the CydA and CydB pathway from the cytoplasmic side to the active site.

Supplementary Table 1. Cryo-EM data collection, refinement and validation statistics.

	Cytochrome *bd*	AD-cytochrome *bd*
**Data collection**		
Microscope	Titan Krios	Titan Krios
Voltage (kV)	300	300
Magnification	29,000x	165,000x
Detector	Gatan K3	Gatan GIF-K2
Data collection software	SerialEM	SerialEM
Electron exposure ($e^{-}/Å^2$)	60	60
Defocus range (μm)	1.2 - 1.8	1.2 - 1.8
Pixel size (Å)	0.82	0.82
**Data processing**		
Number of micrographs	9543	3740
Final particle images	270,938	158,610
Symmetry imposed	C1	C1
Map resolution (Å)		
FSC 0.143 threshold	2.79	2.87
**Refinement**		
Initial model used (PDB code)		
Map sharpening B factor ($Å^2$)	-120	
Map correlation coefficient	0.89	
Mean CC for ligands	0.86	
Model composition		
Non-hydrogen atoms	6,232	
Protein residues	793	
Ligands	HDD: 1	
	HEB: 2	
R.M.S. deviations		
Bond lengths (Å)	0.032	
Bond angles (°)	2.370	
Validation		
MolProbity score	1.86	
Clashscore	6.64	
Poor rotamers (%)	0.00	
Ramachandran plot		
Favored (%)	91.74	
Allowed (%)	8.26	
Disallowed (%)	0.00	
Cβ outliers (%)	0.00	

Supplementary Table 2. Primers used in molecular cloning

pMV261-cydAB-F	5'- tacttccaatccaatgctatggacgctctggacttatc-3'
pMV261-cydAB-R	5'- ttatcccacccaaatggctcgaccgcctggacaatc-3'
pMV261-F	5'-atttgggtgggataaaagc-3'
pMV261-R	5'-gcattggattggaagtagg-3'

Supplementary References

1. Kumar S. *et al*. MEGA X: molecular evolutionary genetics analysis across computing platforms. *Mol. Biol. Evol.* **35**,1547-1549 (2018).
2. Ivica Letunic, Peer Bork. Interactive Tree Of Life (iTOL) v4: recent updates and new developments, *Nucleic Acids Res.* **47**,256–W259(2019).
