# Supplementary material for: Cryo-EM structure of mycobacterial cytochrome bd reveals two oxygen access channels
Source: Nat Commun. 2021 Jul 30;12:4621. doi: 10.1038/s41467-021-24924-w (PMC8324918; doi:10.1038/s41467-021-24924-w)
Supplement: Supplementary file 2 — Reporting summary [file 41467_2021_24924_MOESM2_ESM.pdf]

nature research

Corresponding author(s): Zihe Rao

Last updated by author(s): Jun 30, 2021

Reporting Summary

Nature Research wishes to improve the reproducibility of the work that we publish. This form provides structure for consistency and transparency in reporting. For further information on Nature Research policies, see Authors & Referees and the Editorial Policy Checklist .

Statistics

For all statistical analyses, confirm that the following items are present in the figure legend, table legend, main text, or Methods section.

n/a	Confirmed	
☐	☒	The exact sample size (*n*) for each experimental group/condition, given as a discrete number and unit of measurement
☐	☒	A statement on whether measurements were taken from distinct samples or whether the same sample was measured repeatedly
☐	☒	The statistical test(s) used AND whether they are one- or two-sided<br>*Only common tests should be described solely by name; describe more complex techniques in the Methods section.*
☒	☐	A description of all covariates tested
☒	☐	A description of any assumptions or corrections, such as tests of normality and adjustment for multiple comparisons
☐	☒	A full description of the statistical parameters including central tendency (e.g. means) or other basic estimates (e.g. regression coefficient) AND variation (e.g. standard deviation) or associated estimates of uncertainty (e.g. confidence intervals)
☒	☐	For null hypothesis testing, the test statistic (e.g. *F*, *t*, *r*) with confidence intervals, effect sizes, degrees of freedom and *P* value noted<br>*Give P values as exact values whenever suitable.*
☒	☐	For Bayesian analysis, information on the choice of priors and Markov chain Monte Carlo settings
☒	☐	For hierarchical and complex designs, identification of the appropriate level for tests and full reporting of outcomes
☒	☐	Estimates of effect sizes (e.g. Cohen's *d*, Pearson's *r*), indicating how they were calculated

Our web collection on statistics for biologists contains articles on many of the points above.

Software and code

Policy information about availability of computer code

Data collection	SerialEM 3.6
Data analysis	MotionCor2 1.2.1, cryoSPRAC 2.9, Relion 3.03, Phenix 1.14, COOT 0.8.9, UCSF Chimera 1.12, PyMol 2.0.1, ResMap, 3DFSC, MEGA X, iTol v5, UCSF Chimera X, GraphPad prime 6.0, OriginPro 2021.

For manuscripts utilizing custom algorithms or software that are central to the research but not yet described in published literature, software must be made available to editors/reviewers. We strongly encourage code deposition in a community repository (e.g. GitHub). See the Nature Research guidelines for submitting code & software for further information.

Data

Policy information about availability of data

All manuscripts must include a data availability statement. This statement should provide the following information, where applicable:

- Accession codes, unique identifiers, or web links for publicly available datasets
- A list of figures that have associated raw data
- A description of any restrictions on data availability

The accession numbers for the 3D cryo-EM density map of Msm bd oxidase without and with bound AD in present study are EMD-30582 and EMD-31302, respectively. The accession number for the coordinates for the Msm bd oxidase without bound AD in this study is PDB: 7D5I.
PDB code: 7D5I https://doi.org/10.2210/pdb7D5I/pdb
EMDB code: EMD-30582 https://www.emdataresource.org/EMD-30582; EMD-31302 https://www.emdataresource.org/EMD-31302

Field-specific reporting

Please select the one below that is the best fit for your research. If you are not sure, read the appropriate sections before making your selection.

[x] Life sciences [ ] Behavioural & social sciences [ ] Ecological, evolutionary & environmental sciences

For a reference copy of the document with all sections, see nature.com/documents/nr-reporting-summary-flat.pdf

Life sciences study design

All studies must disclose on these points even when the disclosure is negative.

Sample size	The sample size of bd complex could be judged approximately by chromatography in Supplementary Fig. 1a/1b. The molecular weight of each components, CydA, CydB and MSP1D1 can be judged by SDS-PAGE.<br>For cryo-EM data collection and processing, sample size was chosen to acquire most reasonable resolution and map features.<br>For oxygen consumption rate (OCR) experiment, sample size was chosen as sufficient to evaluate the average OCR and its standard deviation.
Data exclusions	No data exclusions were radomly into experimental groups.
Replication	Each step of the experiments is repeatable. The whole protein complex can be purified every time with good homogeneity. The quality of cryo-EM maps was evaluated based on the gold-standard FSC 0.143 criteria. In oxygen consumption rate experiment (Supplementary Fig. 1e), data shows averages of four technical replicates. Therefore, we confirmed all attempts at replication were successful.
Randomization	Samples were allocated randomly into experimental groups.
Blinding	Investigators were blinded to group allocation during data collection and/or analysis

Reporting for specific materials, systems and methods

We require information from authors about some types of materials, experimental systems and methods used in many studies. Here, indicate whether each material, system or method listed is relevant to your study. If you are not sure if a list item applies to your research, read the appropriate section before selecting a response.

Materials & experimental systems

n/a	Involved in the study	
[x]	[ ]	Antibodies
[x]	[ ]	Eukaryotic cell lines
[x]	[ ]	Palaeontology
[x]	[ ]	Animals and other organisms
[x]	[ ]	Human research participants
[x]	[ ]	Clinical data

Methods

n/a	Involved in the study	
[x]	[ ]	ChIP-seq
[x]	[ ]	Flow cytometry
[x]	[ ]	MRI-based neuroimaging
